# Interaction of human gut microbiota and local immune system in progression of colorectal adenoma (MIMICA-1): a protocol for a prospective, observational cohort study

**DOI:** 10.3389/fonc.2024.1495635

**Published:** 2025-01-06

**Authors:** Jurate Valciukiene, Egle Lastauskiene, Aida Laurinaviciene, Matas Jakubauskas, Marius Kryzauskas, Ruta Barbora Valkiuniene, Renaldas Augulis, Ausra Garnelyte, Justinas Kavoliunas, Ugne Silinskaite, Tomas Poskus

**Affiliations:** ^1^ Clinic of Gastroenterology, Nephro-Urology, and Surgery, Institute of Clinical Medicine, Faculty of Medicine, Vilnius University, Vilnius, Lithuania; ^2^ Institute of Biosciences, Life Sciences Center, Vilnius University, Vilnius, Lithuania; ^3^ National Center of Pathology, Vilnius University Hospital Santaros Klinikos, Vilnius, Lithuania; ^4^ Department of Pathology, Forensic Medicine and Pharmacology, Institute of Biomedical Sciences, Faculty of Medicine, Vilnius University, Vilnius, Lithuania; ^5^ Faculty of Medicine, Vilnius University, Vilnius, Lithuania

**Keywords:** gut microbiota, bacterial dysbiosis, intestinal immunity, colorectal polyps, colorectal adenoma, carcinoma in situ, adenocarcinoma, carcinogenesis

## Abstract

**Introduction:**

The current understanding of colorectal carcinogenesis is based on the adenoma-carcinoma sequence, where genetics, intestinal microbiota changes and local immunity shifts seem to play the key roles. Despite the emerging evidence of dysbiotic intestinal state and immune-cell infiltration changes in patients with colorectal adenocarcinoma, early and advanced adenoma as precursors of colorectal cancer, and carcinoma *in situ* as the following progression, are rather less studied. The newly colon-site adapted AI-based analysis of immune infiltrates is able to predict long-term outcomes of colon carcinoma. Though it could also facilitate the pathologic evaluation of precancerous lesion’s potential to progress. Therefore, the purpose of this prospective cohort study (MIMICA-1) is, firstly, to identify the intestinal microbiota and immune infiltration patterns around the normal bowel tissue, early and advanced adenoma, carcinoma in situ, and adenocarcinoma, and secondly, to analyze the immune – microbiome interplay along the steps of conventional colorectal tumorigenesis.

**Methods and analyses:**

This study aims to prospectively recruit 40 patients (10 per group) with confirmed colorectal dysplasia undergoing endoscopic polypectomy, endoscopic mucosal resection for colorectal small (≤1cm), and large (>1cm) adenoma or carcinoma in situ, or biopsy and subsequent colon resection for invasive colorectal cancer, and 10 healthy patients undergoing screening colonoscopy. Stool samples will be collected prior to bowel preparation for the analysis of fecal (luminal) microbiota composition. Biopsy specimens will be taken from the terminal ileum, right colon, left colon, and a pathological lesion in the colon (if present) to assess mucosa-associated microbiota composition and intestinal immunity response. DNA will be extracted from all samples and sequenced using the Illumina MiSeq platform. Unifrac and Bray-Curtis methods will be used to assess microbial diversity. The intestinal immune system response will be examined using digital image analysis where primarily immunohistochemistry procedures for CD3, CD8, CD20 and CD68 immune cell markers will be performed. Thereafter, the count, density and distribution of immunocompetent cells in epithelial and stromal tissue compartments will be evaluated using AI-based platform. The interaction between the microbial shifts and intestinal immune system response in adenoma-carcinoma sequence and the healthy patients will be examined. In addition, fecal samples will be explored for gut microbiota’s composition, comparing fecal- and tissue-derived bacterial patterns in healthy gut and along the adenoma-carcinoma sequence.

**Discussion:**

We hypothesize that changes within the human gut microbiota led to detectable alterations of the local immune response and correlate with the progression from normal mucosa to colorectal adenoma and invasive carcinoma. It is expectable to find more severe gut immune infiltration at dysplasia site, though analyzing invasive colorectal cancer we expect to detect broader mucosa-associated and luminal microbiota changes with subsequent local immune response at near-lesion site and possibly throughout the entire colon. We believe that specific compositional differences detected around premalignant colorectal lesions are critically important for its primary role in initiation and acceleration of colorectal carcinogenesis. Thus, these microbial patterns could potentially supplement fecal immunohistochemical tests for the early non-invasive detection of colorectal adenoma. Moreover, AI-based analysis of immune infiltrates could become additional diagnostic and prognostic tool in precancerous lesions prior to the development of colorectal cancer.

**Registration:**

The study is registered at the Australian New Zealand Clinical Trials Registry (ACTRN12624000976583) https://www.anzctr.org.au/.

## 1 Introduction

Colorectal cancer (CRC) represents the third most common cancer and is the second-leading cause of cancer-related deaths worldwide (1, 2). The exact etiology of CRC remains unclear, although several studies have focused on the evaluation of the mechanisms involved. Most CRC cases (approximately 90%) occur sporadically (not related to the genetics of family with history of the disease), and several lifestyle factors including obesity, diet with a high content of fat and/or red and/or processed meat, environmental pollutants, cigarette smoking, and alcohol abuse, have been associated with CRC development and progression (2–4). Furthermore, there is emerging evidence that patients with CRC display significant alterations in gut microbiota, mainly characterized by an increase in opportunistic pathogens (e.g., *Enterococcaceae*, *Campylobacter*) (5, 6) and a decrease in butyrate-producing bacteria, including *Bifidobacteria*, *Roseburia* and *Faecalibacterium prausnitzii* (5–8). Overall, these alterations in the gut microbiome have been proposed to play an important role in tumor formation and progression (8–11).

As the initial genetic composition of all cells of a single human is identical, it is fairly questionable why the cells of the distal small bowel have 200 times reduced risk of cancer, when compared to the proximal large bowel (12–14). On the one hand, embryological development of human gastrointestinal tract through foregut, midgut and hindgut structures may play the most significant role. Whilst the right and proximal two-thirds of the transverse colon are midgut structures like the majority of the small intestine, the remaining distal colon and rectum are embryologically formed from the hindgut (15). Therefore, structurally, functionally and, in general, ontogenetically small and large intestines are different organs. Nevertheless, the role of continuous presence of microbes and decreasing immune cell infiltration towards distal large bowel cannot be diminished (16). Particularly, the composition of site-specific (luminal, and especially, mucosa-associated) bowel microbiota, along with the changes in local immune response have been discovered to have direct and indirect implications on anti- and procarcinogenic act in the gut (16–18).

With the expansion of research in the field of the human microbiome and immunomodulatory effect of microbiota, the immune – microbiome interplay in CRC began to be more precisely assessed (17, 18). Tumor – infiltrating lymphocytes (TILs) and their distributions within tumor microenvironment (TME) compartments have been reported as potential prognostic and predictive biomarkers in various cancer types, including CRC (19). The count, density, and distribution of immunocompetent cells (e.g. CD3, CD8, CD20, CD68 and CD163, FOXP3; CD8/CD20 Immunogradient or Immunoscore) in epithelial and stromal compartments are being used for the evaluation of host local-intestinal immune system response in cancerous colorectal tissues (20–22). Studies of local gut immunity highlight its practical importance in terms of CRC treatment (immunotherapy), as well (23). Currently developed digital image analysis (DIA) tools and spatial analytics increase the accuracy and precision of TILs measurements. It can also enable the analysis of subvisual cell distribution patterns that all together improve the informative power of immune response profiling and the extraction of novel indicators (22, 24). Moreover, recently the prognostic models that integrate local immune response and histopathological features have shown additional value predicting CRC outcomes (20, 21, 25). Similarly, the combinatorial analysis of the immune system – microbiome interaction in the context of pathology and genetic alterations is likely to reveal new insights into adenoma – carcinoma development (26–28).

Several studies found that changes in the gut microbiota manifest in disease progression as a result of epigenetic modification in the host, which in turn influences the differentiation and function of immune cells adversely (27, 28). CRC was also shown to be associated with higher levels of oncogenic mutations, such as K-ras, COX2, c-MYC and p53 (27–29). Accumulation of these genetic changes within colorectal epithelial cells is a basis of the current understanding of adenoma-carcinoma sequence. However, the interplay between the three components – genetics, immunity, and gut microbiota compositional shifts – are not yet studied sufficiently (30). To the best of our knowledge, our study would be one of the first to concurrently examine the relation of microbial, histologic, and local immune changes in the progression of colorectal adenoma to cancer.

### 1.1 Aims of the study

We expect to advance the knowledge of CRC pathogenesis by studying the changes of the microbiota structure around early, advanced adenoma, carcinoma *in situ* and CRC, and comparing them to the microbiota of the distal small bowel wall and the stool microbiota of the same person and other healthy patients (healthy controls). Apart from microbiota analysis, we also aim to determine the immune infiltration patterns of the area around adenoma and carcinoma and compare them to the healthy segments of the same person’s distal small and large bowel, as well as to the bowel wall immune-cell infiltrates of the healthy controls. The latter will be performed by employing the AI-based digital image analysis, which is currently being used in predicting colorectal cancer outcomes (20–22). In addition to this, in our study the AI-based DIA will be used to describe premalignant dysplastic colorectal lesions and normal bowel wall structures. Hopefully, this deep-learning image processing technique could be a promising tool for the pathologic evaluation of precancerous lesion’s capacity to progress.

Hypothetically, if relevant differences were found between microbial composition and immune changes, the samples collected during this study would be further explored to evaluate whether they correspond to the growing collection of genetic abnormalities leading to the development of CRC.

Sample collection is another challenging step in human gut microbiota studies. Many studies on the gut microbiota, including those related to CRC, are conducted using fecal samples, since its collection is an easy, non-invasive and repeatable procedure (31, 32). Thus, while fecal samples represent a powerful strategy for finding diagnostic and prognostic biomarkers, tissue samples (from colonic mucosa and dysplastic changes) may be more valuable to disentangle the physiopathology of CRC (6, 33, 34). Accordingly, in our study, we aim to investigate the difference between fecal- and tissue-derived gut bacterial compositions and their role in human bowel microbiota studies in order to increase sampling accuracy and applicability.

In addition, while performing this study, we would advance the knowledge on the dynamics of the normal bowel microbiota and on the normal local immune system of the distal small and large bowel.

### 1.2 Objectives and tasks of the study

The primary objective of the study is to identify the gut microbiota and immune infiltration patterns around normal bowel tissue, early adenoma, advanced adenoma, carcinoma *in situ* and invasive carcinoma.

The secondary objectives of MIMICA-1 are to investigate whether intestinal microbiota correlates with local immune response, and to compare mucosa-associated and fecal-derived microbiota compositional changes in healthy patients and along the adenoma-carcinoma sequence.

The aim will be achieved by performing the following tasks within the study:

1. To evaluate the normal microbiota of the distal small bowel, the right side of the large bowel and the left side of the large bowel and correlate the microbiota of the bowel wall to the stool microbiota;
2. To determine the microbiota of the early (<1cm) adenoma, advanced (>1cm) adenoma, carcinoma *in situ* and invasive carcinoma and to compare it to the microbiota of the normal bowel as well as in the healthy segments of the small and large bowel;
3. To study the normal immune infiltration (in the form of count, density and distribution of immunocompetent cells in epithelial and stromal tissue compartments) of the normal small and large bowel and to correlate it to the normal bowel wall and stool microbiota;
4. To compare the normal immune infiltration to the immune infiltration around the early (<1cm) adenoma, advanced (>1cm) adenoma, carcinoma *in situ* and invasive carcinoma of the large bowel;
5. To identify, whether changes in microbiota and immune environment interrelate and correlate with the progression of the dysplastic changes within the epithelium of the large bowel;
6. To collect and contain colon mucosal biopsies in a biobank for further examination of intestinal epithelial cell oncogenic mutations and its correlation between gut microbiota’s compositional changes and the response of the intestinal immune system in the healthy small bowel and colon mucosa, and across adenoma-carcinoma sequence (in simple CR adenoma (< 1 cm); advanced CR adenoma (≥ 1 cm), Ca *in situ* and CRC).

## 2 Methods and analysis

### 2.1 Study design

#### 2.1.1 Setting

The present trial (MIMICA-1) is an open prospective observational cohort study conducted in a tertiary high volume expert Center for Abdominal and Onco-Surgery and Hepatology, Gastroenterology and Dietetics Center at the Vilnius University Hospital Santaros Klinikos, Vilnius, Lithuania.

#### 2.1.2 Approvals

The study protocol was reviewed and approved by an independent Vilnius Regional Bioethics Committee (Lithuania) in April 2022 (internal No.: 2022/4-1422-902) and registered at web-based Australian New Zealand Clinical Trials Registry (ACTRN12624000976583) https://www.anzctr.org.au/ in July 2024. No significant protocol changes or deviations will be allowed without documented approval.

**Figure 1:**
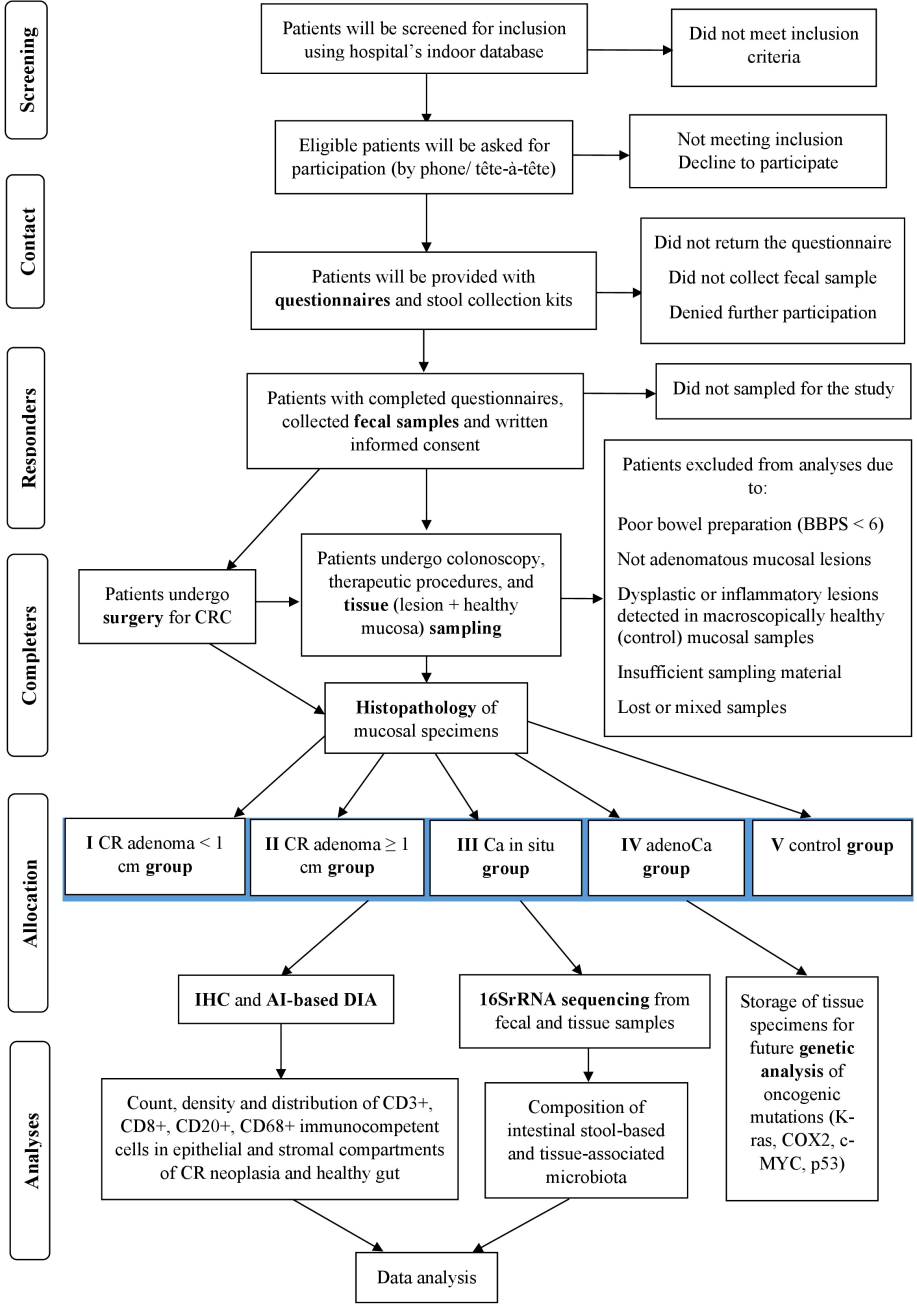
Flow diagram of the study. CR, colorectal; Ca in situ, carcinoma *in situ* (carcinoma in adenoma/intramucosal carcinoma); adenoCa, adenocarcinoma; IHC, immunohistochemistry; DIA, digital image analysis; BBPS, Boston Bowel Preparation Scale.

The study will be carried out in accordance with the principles of the Declaration of Helsinki and Strengthening the Reporting of Observational Studies in Epidemiology (STROBE) statement (35). A STROBE checklist (**Supplementary Table 1**) and a SPIRIT checklist (**Supplementary Table 2** and **Figure 1**) are attached.

#### 2.1.3 Consent

Patient informed written consent for participation will be obtained by the specialist or project’s researcher prior to inclusion in the study. A member of the team will inform patients of the objectives and methodology of the study and patients will be given a written patient information sheet. Patients consent to have their stool samples and intestinal tissue specimens collected and subsequently analyzed for microbiota composition and local immune infiltration. Additionally, participants consent to have collected tissue specimens stored at a biobank for up to 5 years for future genetic analysis of specific oncogenic mutations affecting the progression of colorectal adenoma. Patients will be able to ask any questions regarding participation in the study. Participants may withdraw or revoke consent at any time without giving explanations and without any prejudice to them. No financial incentives will be provided to any of the participants.

### 2.2 Study population

Adult patients (from 18 years old) who will be referred for a colonoscopy either through the bowel cancer screening program, or as a part of a surveillance program, or due to symptoms and diagnosed with histologically confirmed colorectal dysplasia (exposure groups) or adult patients without any polypoid lesions found during the colonoscopy (control group). It is expected that most of the participants will be referred due to iron deficiency anemia, altered bowel habits, weight loss, rectal bleeding, planned polypectomy, CRC-associated family history, abnormal imaging, or polyp surveillance.

#### 2.2.1 Eligibility criteria

##### 2.2.1.1 Inclusion criteria for exposure groups

1. Patients with histologically confirmed dysplastic colorectal lesion: early and advanced-adenoma, carcinoma *in situ* or adenocarcinoma. Advanced adenomas are defined as those with high-grade dysplasia, villous or tubulovillous histology, or a diameter ≥1 cm;
2. Adult patients (≥18 years);
3. Written informed consent.

##### 2.2.1.2 Inclusion criteria for control (healthy) group

1. Healthy patients (without any polypoid lesions found during screening colonoscopy);
2. Adult patients (≥18 years);
3. Written informed consent.

##### 2.2.1.3 Exclusion criteria

1. Patients under the age of 18 years;
2. Confirmed serrated (sessile serrated (SSA), traditional serrated adenomas (TSA)) or hyperplastic polyps or non-polypoid lesions;
3. Signs of colorectal tumor obturating the lumen of the bowel which would limit complete colonoscopy;
4. Suffer from other gastrointestinal tumors;
5. Pregnancy;
6. Previous colon resection;
7. History of surgery disrupting gastrointestinal tract integrity;
8. History of inflammatory bowel disease (ulcerative, Crohn’s, radiation-induced, or infectious colitis or other previous chronic inflammatory illnesses);
9. Familial adenomatous polyposis (FAP) or other hereditary colon syndromes;
10. Clinically significant immunodeficiency;
11. Evidence of infection;
12. During the last year patient had:
  - suffered from *Cl. difficile* colitis or was a carrier of *Cl. difficile*; suffered from salmonellosis or other gastrointestinal infection;
  - a long-term (> 6 months) use or recently completed therapeutic antibiotic course within the last month;
  - corticosteroid and/or immunosuppressant therapy;
  - received chemo- or radiation therapy in the abdomen and/or pelvis chemotherapy;
  - regular use (> 3 months) of pre-/pro-/(sin)biotics and/or statins;
  - a long-term (> 6 months) use of proton pump inhibitors;
13. Patients who cannot undergo colonoscopy on time and cannot cooperate fully.

#### 2.2.2 Selection and recruitment

Patients will be screened for inclusion criteria using hospital’s indoor database, the ICD-10 codes and the schedule of elective screening and diagnostic colonoscopies, endoscopic polypectomies, and surgery for CRC (laparoscopic colon resections, in particular). Preliminary selected patients will be contacted, double-checked for inclusion, and asked for participation (by phone or tête-à-tête). Agreed patients will be provided with health questionnaires and stool collection kits in person or by mail. The overall study design is depicted in **Figure 1**.

#### 2.2.3 Questionnaires and physical evaluation

The personal information of the subjects will be conducted through the hospital’s indoor database and advanced health information questionnaires. Patients will be provided with questionnaires containing questions on demographics, anthropometric data (height, weight, body mass index (BMI) and waist circumference), health history, gastrointestinal disorders and interventional procedures, bowel symptoms, co-morbidities (including tobacco and alcohol consumption), use of certain medication (as antibiotics, probiotics, statins, proton pump inhibitors, corticoids and immunomodulators), dietary and ecological habits and possible microplastic intake. Physical evaluation will be assessed by the anesthesiologist on the procedure day.

#### 2.2.4 Sample size calculation

Since there is no clinical trial assessing the composition of intestinal microbiota; the count, density and distribution of immunocompetent cells in epithelial and stromal compartments, and the interrelations of both along every step of conventional adenoma-carcinoma pathway and healthy colon, endpoints of the study are currently unable to be determined. Thus, the sample size is estimated based on the data from a previous clinical trial (36), where 10 participants per group had 80% power at an alpha level of 0.05 and beta level of 0.2 to detect significant differences. The sample size in this study was calculated statistically by G*Power 3.1.9.4. Considering the expected withdrawal of participants during the intervention, we plan to recruit from 10 to 15 participants per group. This results in minimum 50 patients, 190 biopsy locations, 570 tissue samples, and a total number of 2280 specimens (staining using 4 antibodies) to be examined histologically and immunohistochemically. Plus 50 fecal and 570 tissue samples to be 16S rRNA sequenced (after DNA extraction) for precise analysis of gut microbiota structure. Overall, despite the inter-individual heterogeneity, with this sample size, differences in gut microbiota composition and local immunity changes are considered to be detectable.

## 3 Interventions

Adult patients who have been referred to a screening/diagnostic/therapeutic colonoscopy and during procedure will be diagnosed with CR polyps, as a common practice of colorectal dysplasia management will undergo (if no contraindications):

- endoscopic polypectomy, in case of simple CR adenoma;
- endoscopic mucosal resection (EMR) or endoscopic submucosal dissection (ESD), in case of advanced, flat/over the fold/right-sided colon CR adenomas or Ca in situ;
- TEM (transanal endoscopic microsurgery) in case of large rectal adenomas or Ca in situ;
- sampling and subsequent colon resection in case of colorectal cancer or unresectable colon polyps.

During the same procedure seven mucosal biopsy specimens from each region (macroscopically healthy right- and left-sided colon, terminal ileum, and pathologic lesion, if present) will be obtained. In order to prevent possible cross-contamination from other parts of the bowel (especially important in tissue specimens being sampled for microbiota compositional analysis), sterile biopsy forceps will be used for each region and each biopsy location. Resected polyps and mucosal biopsy specimens from unaffected bowel regions will be sent to histological, IHC, DNA sequencing examination and further genetic testing for specific oncogenic mutations (in the next trial). In terms of the study, no further visits to the hospital will be required.

### 3.1 Data collection and management

Assessment of participants will be conducted at the pre-procedure (-t1), procedure (0) and post-procedure (t1-t4) timepoints. The detailed schedule of assessments is depicted in **Table 1**. The primary outcomes will be the differences in the microbiota composition and immune infiltration in different localizations of the tumor and different steps of mucosa dysplastic lesions compared to healthy controls. The secondary outcomes include correlation analysis of gut microbial diversity, intestinal immunity variables and the grade of colorectal dysplasia assessed for the study.

**Table 1:** Study schedule of enrolment, interventions, and assessments.

	STUDY PERIOD						
	Enrolment	Allocation	Examination and analysis period				Close-out
TIMEPOINT	*-t_1_*	0	*t_1_*	*t_2_*	*t_3_*	*t_4_*	*t_x_*
ENROLMENT:							
Eligibility screen	X						
Informed consent + filling the questionnaire	X						
Allocation		X					
INTERVENTIONS:							
Collection of fecal samples	X	X					
Colonoscopy + endoscopic polypectomy (or biopsy) and normal gut tissue sampling or right hemicolectomy and gut tissue sampling	X		X				
ASSESSMENTS AND DATA ANALYSIS:							
Pathologic investigations: HE and IHC	X	X	X				
16S rRNA gut tissue- and fecal-derived microbiota sequencing			X	X	X	X	
AI-based digital image analysis			X	X	X	X	
Qualitative data analysis based on the questionnaires		X	X				
Data analysis on intestinal immune infiltration variables: count, density, and distribution of CD3, CD8, CD20, CD68 cells in epithelial and stromal compartments				X	X	X	X
Data analysis on gut microbiota compositional changes in tissue and stool samples: relative abundance, α- and β-diversity, etc.				X	X	X	X
Correlation analysis of gut microbiota and immune response variables in different site of the colon along the adenoma-carcinoma sequence						X	X

X, mark the appropriate period for the enrollment, interventions and other activities during the project

#### 3.1.1 Pre-procedure

The collection of feces will be carried out prior to bowel preparation for colonoscopy or surgery (from 2 weeks until the planned procedure) at the residence. That way extensive alterations due to bowel cleansing in (only partially constant) individual intestinal luminal microbiota could be avoided. Briefly, participants will be instructed to, firstly, pass stool on the tray, secondly, dig up 1-3 spoons of stool and, thirdly, insert it into a sterile screw cap container. For reducing the change of microbiota composition in the stools, the liquid preservative will be added in each container sent to the preliminary participants. Patients will be asked to bring stool samples to the researcher at the hospital on the procedure day. Those with completed questionnaires, collected stool samples and written informed consent will eventually be enrolled in further study.

#### 3.1.2 Procedure

Highly experienced endoscopists, with proven adenoma detection rate of >30% will perform the colonoscopy, endoscopic polypectomy (if needed) and tissue sampling intended for the study. In case of CRC or endoscopically unresectable polyps, experienced abdominal surgeons specializing in colorectal surgery will take over the sampling procedure, as well. Patients will undergo simple polypectomy, endoscopic mucosal resection (EMR), endoscopic submucosal dissection (ESD), transanal endoscopic microsurgery (TEM) or surgery for CRC depending on the localization, size and histology of the lesion found. Procedures are scheduled as a common management of a colorectal neoplasm and will not be affected by patient’s preliminary enrolment in the study. During colonoscopy or colon resection the mucosal biopsy specimens from the polypoid lesion and the healthy gut (macroscopically healthy right- and left-sided colon and terminal ileum) will be obtained using disposable biopsy forceps (separate for each segment) to avoid bacterial contamination. The tissue sampling plan includes a) tissue of the terminal ileum; b) tissue of the right-sided colon (cecum, ascending colon, right colic flexure); c) tissue of the left-sided colon (descending colon, sigmoid colon and the rectosigmoid junction); and d) tissue of the lesion (if present). In case of small adenomas (<1 cm size), sampling is performed near the lesion, since multiple biopsies directly from the small tumor may result in artificial material fragmentation, at the same time raising the risk of histological misdiagnosis. Hyperplastic polyps and non-polypoid lesions are not being sampled, nor resected during colonoscopy in terms of the study. If multiple diverticula are present, normal tissue of the colon will be sampled at the least affected site. More than ten locoregional polyps are considered as polyposis and are not further investigated under this trial. Otherwise, while up to ten polyps are detected in the same colon region, the largest is being picked for sampling and subsequent investigation. Inflammatory or pseudo-polyps are not adenomatous lesions, thus are not considered eligible for the study. Bowel preparation should be evaluated with at least 6 out of 9 points according to the Boston Bowel Preparation Scale (BBPS), otherwise precise detection and evaluation of dysplastic mucosal lesions cannot be ensured. Those patients who underwent colonoscopy and have been evaluated by less than 6 points according to BBPS will not be included in further study. Resected or sampled polyps should be described using the Paris and NICE classifications. Each stool and tissue sample will be encoded according to localization of the colon and the type of examination it is dedicated for.

#### 3.1.3 Post-procedure

The pseudonymized stool and tissue samples will be labelled with unique study ID and transferred to the hospital’s microbiological laboratory or biobank where they will be frozen at the − 80°C refrigerator for later use. A part of intestinal tissue samples will be assessed immediately for histological evaluation and immunohistochemically for AI-based DIA. Stool samples and mucosal biopsies will be simultaneously examined for fecal and mucosa-associated microbiota. Thereafter, data analysis on microbial composition and its alterations in both fecal and gut tissue specimens will be performed, compared, and correlated. Another part of the collected colon mucosal samples will be contained in a biobank for future study on intestinal epithelial cell oncogenic mutations and its interrelation with intestinal immunity and gut microbiome in the light of colorectal carcinogenesis.

##### 3.1.3.1 Histopathological examination and further allocation

Biopsy specimens (resected polyps and samples of normal colonic and ileil mucosa) will be fixed in 10% buffer formalin 24-48 hours at room temperature, processed and embedded in paraffin (FFPE). The FFPE sample sections will be cut at 3 μm thickness and mounted on positively charged slides. All slides will be stained with hemotoxylin-eosyn (HE) for the accurate evaluation of polyps and healthy colon histology.

Patients with non-adenomatous mucosal and sessile serrated lesions will be eliminated from the study. Only patients with histopathologically confirmed colorectal adenoma (small/large), carcinoma *in situ* and adenocarcinoma are being selected for further examination and become study participants. These patients will be divided into 4 exposure groups according to the CR neoplasia size and the dysplasia level, and 4 self-control groups (patient serves as his own control), and the general control group V as an overall healthy patients’ control (**Table 2**). Patients with macroscopically normal but histologically confirmed adenomatous or non-adenomatous mucosal lesions in control-site biopsies will not be enrolled in further examination and so will be eliminated from the study.

**Table 2:** Histology- and size-based allocation of participants.

Exposure	Control
Group I (N=10): endoscopically removed and histopathologically confirmed up to 1 cm sized (low-grade dysplasia) tubular/tubulovillous/villous adenoma;	Control group 1: biopsy samples from group I patients’ terminal ileum, right- and left-sided healthy colon;
Group II (N=10): endoscopically removed and histopathologically confirmed 1 cm or greater (low- or high-grade dysplasia) tubular/tubulovillous/villous adenoma;	Control group 2: biopsy samples from group II patients’ terminal ileum, right-and left-sided healthy colon;
Group III (N=10): endoscopically removed/sampled and histopathologically confirmed Ca *in situ* (high-grade dysplasia);	Control group 3: biopsy samples from group III patients’ terminal ileum, right- and left-sided healthy colon;
Group IV (N=10): endoscopically sampled and histopathologically confirmed invasive adenocarcinoma;	Control group 4: biopsy samples from group IV patients’ terminal ileum, right- and left-sided healthy colon;
Control group V (N=10): patients, without any polypoid lesions found during colonoscopy (mucosal biopsies from their terminal ileum, right- and left-sided healthy colon).	

##### 3.1.3.2 Immunohistochemistry and AI-based digital image analysis of intestinal mucosal tissue

Immunohistochemical staining will be performed using the Roche Ventana BenchMark ULTRA (Ventana Medical Systems, USA) automated slide stainer. Monoclonal antibodies against B lymphocytes (CD20), T lymphocytes (CD3), cytotoxic T-lymphocytes (CD8) and macrophages (CD68) will be used.

HE, CD3, CD8, CD20, CD68 slides will be scanned using a ScanScope XT Slide Scanner (Leica Aperio Technologies, CA, USA) or an Aperio 18 AT2 Slide Scanner (Leica Microsystems, Wetzlar, Germany) with 20x magnification (0.5 μm resolution). Digitized whole-slide images will be archived in a pathology image database ImageScope (version 11.1.2.752, Leica Biosystems, Chicago, USA), then transferred to a DIA platform HALOTM (version 2.2.1870, Indica Labs, New Mexico, USA).

The intestinal immune system response will be examined using digital pathology image analytics. Primarily, IHC procedures for the visualization of the immune cell markers (CD3, CD8, CD20 and CD68) will be developed and optimized to fit the requirements of DIA. Multiplex IHC will be used to enhance the analysis where both cell populations with specific biological properties and their spatial interactions at the cellular and regional level in tissue microenvironment are included. Then, digital image processing procedures will be established for the robust immune cell infiltrate quantification and analysis of spatial distribution patterns in the intestinal tissues. Analysis of cell population (CD3, CD8, CD20, CD68) will be completed by employing HALO Multiplex IHC module. The methodology that combines DIA, artificial intelligence tools and based on explicit rules will be employed.

The analysis workflow follows: 1) the training of artificial intelligence-based HALO AI classifier to segment the intestinal tissues into classes (stroma, non- malignant epithelium, malignant epithelium etc.); 2) the detection and quantification of immune cell counts/densities in epithelial and stromal compartments. Finally, immune cell population distribution and interaction patterns will be correlated to the composition of the gut microbiota and pathology features. The findings will enable us to generate informative combinatorial models to achieve more precise prediction of CRC development.

##### 3.1.3.3 16S rRNA gene sequencing for microbiota examination from tissue and fecal samples

Tissue samples including lesion (or para-lesion in case of small (<1cm) adenoma), right- and left-sided normal appearing colon and terminal ileum mucosa will be obtained from the participants during colonoscopy or surgery. Intestinal mucosal specimens will be frozen and stored in the − 80°C refrigerator for later use. Similarly, fecal samples of patients will be frozen and stored at − 80°C immediately after they have been received in the study centre.

The total DNA of frozen samples will be extracted by mechanical and enzymatical cell lysis. DNA integrity and concentration will be assessed by the NanoDrop ND-1000 spectrophotometer (Thermo, USA). The bacterial 16S rRNA V3–V4 regions will be amplified via polymerase chain reaction (PCR), and high-throughput sequencing will be conducted on an Illumina platform. Raw reads will undergo denoising and preprocessing utilizing the tools provided by the Quantitative Insights Into Microbial Ecology 2 (QIIME2) software pipeline platform using default settings, unless specified otherwise. The DADA2 workflow will be utilized to construct the amplicon sequence variants (ASV) table, and sequences will be aligned to build a phylogenetic tree. Low abundant sequences will be excluded from analysis. Taxonomic analysis will be conducted with a Naïve Bayes Classifier, leveraging on the SILVA 138.1 database.

## 4 Data and statistical analysis

The questionnaires and other measured data during the visit will be collected on paper and then transcribed to a secure electronic version on a locked office computer. The paper version of the data will be locked in a bookcase. Only the investigators running the study will have access to the final study dataset.

The obtained data will be analyzed by the SPSS software V26.0 (IBM, USA). The count, density, and distribution of immunocompetent cells (CD3, CD8, CD20, CD68) in epithelial and stromal compartments of adenoma, Ca in situ, CRC and the healthy controls will be performed. Here, continuous data with normal distribution will be expressed as mean ± standard deviation (SD), while data that is not normally distributed as median and range. Categorical data (especially from the questionnaires) will be expressed as the number of cases (n) and percentage (%). Kruskal-Wallis Test followed by Dunn’s Test will be employed for multiple pairwise comparisons of differences between two and more groups.

As for statistical analysis of microbial data, relative abundances, alpha and beta diversity will be performed. Principal Coordinates Analysis (PCoA) will be executed after calculating Bray-Curtis dissimilarity and UniFrac distances between samples. Beta diversity analyses will involve the application of Hellinger transformation to account for the compositional nature of microbiome data. Cluster analysis will be performed using Analysis of Similarities (ANOSIM) to assess the significant differences in microbial community composition between sample groups. Redundancy analysis will be used to identify factors that influence the structure of the microbiome. Alpha diversity will be calculated through the Shannon index, Simpson index, and Chao1 metrics after appropriate rarefaction. For the identification of differences in specific taxa between groups, ANCOM and LEfSe analysis will be conducted.

The correlation between the gut bacterial composition and intestinal immune system response in adenoma-carcinoma sequence, and the healthy patients will be examined. Furthermore, fecal samples will be explored for gut microbiota alterations, comparing fecal- and tissue-derived bacterial compositions in healthy gut and along the steps of conventional colorectal carcinogenesis. The results will be regarded as statistically significant if p values < 0.05. In case of a relevant number of missing values, multiple imputation or pattern mixture models will be used.

## 5 Discussion

### 5.1 General insights

Recent studies have increasingly addressed the role of gut microbiota in CRC. Multiple trials have reported a dysbiotic state in fecal-derived and tissue-associated microbiota of colorectal adenoma and CRC patients (36–40). Other studies have highlighted the importance of certain gut bacteria, such as *Fusobacterium nucleatum*, *enterotoxigenic Bacteroides fragilis (ETBF)*, and *colibactin-producing Escherichia coli (EPEC)*, in the onset and progression of CRC (7, 41–45).

Our study aims to test the hypothesis that changes within the human gut microbiota lead to detectable alterations of the local immune response and correlate with the progression from normal mucosa to colorectal adenoma and invasive carcinoma.

The primary objective of the study is to determine the composition of gut microbiota and local intestinal immune infiltrates in healthy gut and along the steps of conventional colorectal carcinogenesis. Secondary objectives include research on the correlation between alterations of gut microbiota and immune infiltration in relation to the severity and size of the colorectal dysplasia, the site of the lesion and the matrix (mucosal tissue or stool) of the sample.

In the outlined study, statistically significant tissue-derived bacterial dysbiosis are likely to appear in the exposure groups of patients with CR adenomas and Ca *in situ* compared to own healthy gut controls and healthy patients’ controls. It is expected to find more severe microbiota’s compositional changes and significantly more expressed intestinal immune response in colorectal cancer vs. adenoma, high-grade vs. low-grade dysplasia; ≥1 cm vs. < 1 cm size polyps; distal vs. proximal colon. CRC-associated bacteria (*F. nucleatum*; *Str. gallolyticus*; *ETBF*; *EPEC* and *E. faecalis*), as studies in the field have observed (7, 42–48), are expected to show higher relative abundance in Ca *in situ* and advanced-adenomas compared to simple adenomas and controls.

The reviewed literature also states that when there is a change in the gut microbiota in a patient with a colorectal neoplasm, it is very difficult to determine whether it is a cause or consequence of colorectal neoplasm (49, 50). We hypothesize that specific gut microbial patterns detected around premalignant lesions compared to healthy gut and healthy patients’ controls are critically important for microbiota’s primary role in the initiation and acceleration of colorectal carcinogenesis. On the contrary, bacterial communities prevailing in carcinoma-linked gut are considered to form chronic inflammation induced secondary alterations, giving the idea of consequential rather than causal relation. Thus, bacterial patterns typical for adenomas could potentially supplement fecal immunohistochemical tests for the early non-invasive detection of precancerous lesions prior to the development of CRC (51–53).

Literature also reports that a change of gut microbiota in the neoplastic tissue is useful to determine passenger bacteria, while a change of gut microbiota in non-neoplastic tissue of the same gut is rather helpful to decide driver bacteria (36, 54–57). Therefore, we aim to analyze gut microbiota’s composition and intestinal immune infiltrates in both lesion and non-lesion tissue of the right-, left-sided colon and terminal ileum, which is hypothetically affected, in all steps of adenoma-carcinoma sequence.

The statistically significant alterations of colon-common bacteria and/or local immunity shifts detected not only in colorectal neoplasia specimens, but also in the normal-looking colon mucosal samples, could perfectly define colorectal dysplasia as a systemic phenomenon affecting the whole colon. While the samples from macroscopically normal terminal ileum comparing healthy patients and patients with colorectal neoplasm will presumably show a more constant structure of gut microbiota and relatively smaller change in immune response during colorectal carcinogenesis. The analysis of ileil samples will undoubtably supplement existing knowledge on the local microbiota composition and extent of mucosal immune infiltration in the small bowel in health and during colorectal adenoma progression to CRC. Here, the main role is shared by the structure of the intestinal wall, local immunity, and bowel transit time.

In addition, the mentioned sampling of the normal-appearing intestinal tissue provides us with a self-control cohort of specimens from the same patient. This additional control group could prevent us from misleading interpretations while comparing gut microbiota composition among various dysplastic lesions, different colon sites, both intra- and interindividually.

Considering all this, MIMICA-1 is one of the pioneering studies to assess the interrelation of microbial, histologic, intestinal immune and oncogenic (in future study) changes in a multidisciplinary manner and in every step of conventional colorectal carcinogenesis. Our study also seeks to elucidate whether and how vast the difference is between fecal- and tissue-derived gut microbiota’s compositions in patients with colorectal neoplasia. As multiple trials show, the examination of mucosa-associated microbiota can provide more specific compositional information compared to stool samples representing luminal microbiota (6, 8, 32–35, 58). While fecal samples are less stable, depending on diet, BMI and behavioral factors, they are still easier to obtain, repeatable, and thus, more suitable for identification of noninvasive diagnostic and prognostic markers in CRC (59, 60). Similarly, this trial is expected to provide evidence for the benefits of more profound understanding of human intestinal microbiota studies, in general, and the role of appropriate sample matrix selection in future studies.

Moreover, the newly colon-site adapted AI-based digital image analysis of immune infiltrates is able to predict long-term outcomes of colon carcinoma (61, 62). Analyzing count, density, and spatial distribution of immunocompetent cells in epithelial and stromal tissue compartments of colorectal adenoma could also become an additional pathologic instrument to predict its further progression to CRC.

### 5.2 Limitations of the study

The single-centre type of this study could be a limit, potentially exposing to recruitment biases. The efforts to avoid selection bias could also be hindered by the fact that sessile serrated lesions as the alternative pathway of colorectal carcinogenesis are not being examined. The study could also be hampered due to the restricted examination of mainly bacterial microbiota composition. The sample size of about 50 participants nevertheless responds to 190 sampling sites, 570 tissue specimens and 50 stool samples being examined for microbiota and local immunity structures, therefore, is not considered small, especially in the light of human gut microbiota studies (63). Due to expected lower incidence of Ca *in situ* lesions in the population, the study has a risk of being limited by the lack of samples in III exposure group. However, in that case the risk could be mitigated by relocating exposure groups and representing adenoma-carcinoma sequence in the form of subsequent dysplasia examination from small (early) and large (advanced) adenomas as initial stages and invasive carcinomas (Ca *in situ* and adenocarcinoma) as the progression of the disease.

### 5.3 Future prospects

Evaluation of gut microbiota’s composition patterns around the CR adenoma and Ca *in situ* could be useful in identifying the primary risk factors for these precancerous conditions, thus possibly preventing adenomas and their genesis to CRC.

Microbiome - immunity axis evaluation may provide more information on adenoma – carcinoma sequence pathogenesis, in general, and could help better identify patients at high-risk of tumor development, at the same time improving the quality of life by predicting and stratifying patients who will benefit from preventing adenomas most.

By integrating data on the gut microbiota, immune responses, and oncogenic mutations, we will begin to obtain the necessary scientific knowledge to optimize microbiota-immune response interactions for effective cancer prevention (dietary interventions and probiotics) (64, 65), and treatment (immunotherapy) (23, 66).

In addition, future research on the expression of oncogenic mutations in adenoma, Ca in situ, CRC and healthy controls is critically important, as well. The interrelation between intestinal microbiota, local immune system response and the expression of oncogenic mutations along the adenoma-carcinoma pathway is key directions for future studies in the field.

## 6 Ethics and dissemination

### 6.1 Confidentiality

All research records associated with the trial will only identify the patient by their initials, date of birth and study number. The patient’s name will not be used in any public report of the study. All study-related information will be safely stored at the study site by the principal investigator. All the data generated from the project will be entered into an excel sheet by three independent researchers of the team. Files will then be double-checked for potential errors by a fourth independent researcher. The digital data will be saved on a dedicated, secured server running on a password protected computer, while paper data including clinical and non-clinical information, protocols, clinical registration forms will be stored in a locked file cabinet in an area with limited access. Participants’ information will be treated following the current European General Data Protection Regulation (2016/679 (GDPR)) (38, 67). Only the principal investigator and authorized individuals will have access to the complete dataset. The data may be used for future research although it should be noted that the anonymized patients will not be reverse-identifiable in the future.

### 6.2 Reporting of the study results

All results obtained from the study will be published in open-access peer-review journals. Patients and the public will not be involved in the development of the research question, outcomes measures or design of the study. Patient care does not differ from the one usually carried out according to the recommendation. Participants will be able to obtain information about the results upon request to the principal investigator. The data will be used in conducting further trials and doctoral research in the field of gut microbiome, intestinal immunity, and host genetics interplay.

The study results will be disseminated among the medical staff by attending relevant conferences and seminars, and the public by organizing talks at regional level. Dissemination activities through Cancer patients’ associations will enable the patients and their relatives to hear more about the study, at the same time increasing awareness of the gut dysbiosis, local immune system response and oncogenic mutations in the development and treatment of colorectal cancer.

Authorship eligibility will follow the criteria established by the SPIRIT guidelines. The study results will be published regardless of the magnitude or direction of effect. Datasets of the microbiota and intestinal immunity analyses will be delivered into an adequate data repository and will be safely stored for at least 5 years after the results are published.
